# Characterization of a Novel α-Neoagarobiose Hydrolase Capable of Preparation of Medium- and Long-Chain Agarooligosaccharides

**DOI:** 10.3389/fbioe.2019.00470

**Published:** 2020-01-29

**Authors:** Chengcheng Jiang, Zhen Liu, Jianan Sun, Xiangzhao Mao

**Affiliations:** ^1^College of Food Science and Engineering, Ocean University of China, Qingdao, China; ^2^Laboratory for Marine Drugs and Bioproducts of Qingdao National Laboratory for Marine Science and Technology, Qingdao, China

**Keywords:** α-neoagarobiose hydrolase, agarase, expression, 3, 6-anhydro-L-galactose, medium and long chain, agarooligosaccharides

## Abstract

α-Neoagarobiose hydrolase plays an important role in saccharification processes of marine biomass. In this study, an α-neoagarobiose hydrolase from *Streptomyces coelicolor* A3(2), designated as ScJC117, was identified, purified, and characterized. It has a sequence of 370 amino acids and belongs to the GH117 family. ScJC117 exhibited good activity under optimal hydrolysis conditions of pH 6.0 and 30°C, where it showed the *K*_m_ and *k*_cat_ for neoagarobiose of 11.57 mM and 0.48 s^–1^, respectively. ScJC117 showed the ability to hydrolyze neoagarooligosaccharides with the polymerization degrees of 2–14. A basis of catalytic activity toward the first α-1,3-glycosidic bond of the neoagarooligosaccharides from the non-reducing end, ScJC117 can be classified as an exo-type α-neoagarobiose hydrolase. These results suggested that ScJC117 could be used in the preparation of odd agarooligosaccharides (especially agaroheptaose-agaroundecaose) and 3,6-anhydro-L-galactose, which has a functional food additive potential. Moreover, ScJC117 can be used for comprehensive utilization of red algae.

## Introduction

Agar, composed of agarose and agaropectin, is one of the major components of red macroalgae (Mouradi-Givernaud et al., 1999). Particularly, agarose is a linear neutral polymer of D-galactose (D-gal) and 3,6-anhydro-L-galactose (L-AHG) units with alternating α-1,3 and β-1,4-glycosidic linkages while agaropectin is made of partially sulfated β-1,3-linked D-gal residues (Smith, 1981). Agarose could be degraded through three methods including acid hydrolysis, acid pre-hydrolysis, followed by enzymatic hydrolysis or enzymatic hydrolysis (Temuujin et al., 2011); the latter needs a combination of different enzymes. Firstly, agarose could be hydrolyzed into neoagarooligosaccharides (NAOSs) by β-agarases (Ekborg et al., 2006; Kim et al., 2010; Benjamin et al., 2013). NAOSs are further hydrolyzed by α-neoagarobiose hydrolases (α-NABHs) to release its non-reducing end L-AHG and remain agarooligosaccharides (AOSs). If NA2 was used as substrate, the products were L-AHG and D-gal (Lee et al., 2009; Jan-Hendrik et al., 2012). L-AHG has cosmetic and pharmacological activities, such as skin whitening and anti-inflammatory (Yun et al., 2013), and Yun et al. suggest that L-AHG can be considered as a new anticariogenic sugar to prevent dental caries (Yun et al., 2017). Due to the high anticariogenic activity of L-AHG, L-AHG could be applicable in various food and dental care products. AOSs could suppress nitric oxide, prostaglandin E2, and pro-inflammatory cytokine production *in vitro* (Tatsuji et al., 2010). Therefore, the α-NABHs not only play an important role in the microbial agar metabolism but also could be applied in food industrial processes for the production of functional monosaccharides and AOSs (Kim et al., 2012, 2013).

The reported α-NABHs widely distributed in a wealth of sources including marine and terrestrial environment (Ekborg et al., 2006). These α-NABHs could be classified into endocellular α-NABH (α-NABHcyt) and exocellular or periplasmic α-NABH (α-NABHext) (Ariga et al., 2014). In 1975, Day et al. discovered the first α-NABH from a marine bacterium, called *Pseudomonas atlantica*, which could produce α-NABH and *p*-nitrophenyl-α-galactosidase at the same time (Day and Yaphe, 1975). Then, an α-NABH produced from *Bacillus* sp. strain MK03 was isolated from the soil of Gifu prefecture in Japan (Suzuki et al., 2002). This α-NABH was found to be capable of simultaneously degrading NA2, neoagarotetraose (NA4), and neoagarohexaose (NA6) into L-AHG and D-gal, agarotriose, and agaropentaose, respectively. In addition, AhgA, derived from the marine bacterium *Zobellia galactanivorans*, is able to hydrolyze neoagarooctaose (NA8) into L-AHG and agaroheptaose (A7) (Etienne et al., 2011). Ha et al. speculated that α-NABHext may have a broad range of substrates formed by extracellular β-agarase. The α-NABHs with smaller amino acids, often serines and alanines rather than phenylalanine or tyrosine, around the substrate binding site may have additional subsites. Moreover, another study assumed that the amino acids around the substrate binding site of α-NABH may decide the range of substrates (Jan-Hendrik et al., 2012).

Moreover, α-NABH was also crucial in the process of agarose degradation. In the agarose degradation pathway, agarose was first degraded into NAOSs (DPs > 4) by an endo-type β-agarase I. The NAOSs are further hydrolyzed to NA2 by a GH50-dependent β-agarase II. Then, α-NABH was used for degrading the NA2 into D-gal and L-AHG. Finally, D-gal was transported into cytoplasm and metabolized through the Leloir pathway to enter the glycolytic pathway (Horváth et al., 2010). Another monosaccharide L-AHG can be metabolized by a two-step enzymatic reaction that was catalyzed by AHG dehydrogenase and AHGA cycloisomerase (Yun et al., 2015). These results indicated that α-NABH was significant for bioconversion of agarose into biofuels or biochemicals.

In this study, a novel α-NABH, designated ScJC117, was characterized and explored for preparation of medium- and long-chain AOSs, such as A7, agarononaose (A9), agaroundecaose (A11), and agarotridecaose (A13). More specifically, we discovered an α-NABH from an agar-utilizing strain *Streptomyces coelicolor* A3(2) that could act on NA8, neoagarodecaose (NA10), neoagarododecaose (NA12), and neoagarotetradecaose (NA14) to produce A7, A9, A11, and A13, while the L-AHG, a rare monosaccharide that has cosmetic and pharmacological activities and can be considered as a new anticariogenic sugar to prevent dental caries, remained. This novel α-NABH can be applied in food industrial processes for the production of functional monosaccharides and AOSs, especially medium- and long-chain AOSs.

## Materials and Methods

### Materials

The yeast extract and tryptone used for the lysogeny broth medium were purchased from Oxoid (Basingstoke, United Kingdom). The NAOSs with different DPs (2–14) used for reaction were purchased from Bz Oligo Biotech (Qingdao, China).

### Bacterial Strains and Culture Conditions

*S. coelicolor* A3(2) was cultivated at 30°C in TSB medium composed of 1.5% (w/v) tryptone, 0.5% soy peptone, and 0.5% NaCl. The *Escherichia coli* strains were grown in LB medium (1% tryptone, 0.5% yeast extract, and 1% NaCl) at 37°C with 50 μg/ml kanamycin (Solarbio, China) when required.

### Sequence Analysis

Function prediction and homology analysis of ScJC117 were performed using the NCBI (National Center for Biotechnology Information, United States) database^1^. Phylogenetic analysis was performed with MEGA version 5.0.

### Cloning, Expression, and Purification of Recombinant ScJC117

Genomic DNA was extracted from *S. coelicolor* A3(2) using a TIANamp Bacteria DNA Kit (Tiangen Biotech, Beijing, China). The primers ScJC117-F 5′-AGTGCGGCCGCAAGCTTCTTTTCGTGCGGAGC-3′ containing a *Hin*dIII site (underlined) and ScJC117-R 5′-AAATGGGTCGCGGATCCATGACCATGCCCGCA-3′ containing a *Bam*HI site (underlined) were designed for amplification of the *sco3481* gene. All of the primers were synthesized by BGI (Beijing, China). The PCR product of *sco3481* was ligated with the pET-28a(+) expression vector by ClonExpress^®^ ultra one step cloning kit (Vazyme, China) and the nucleotide sequence of the inserted gene fragment was confirmed by sequencing (BGI, Beijing, China). The recombinant vector was then transformed into competent *E. coli* BL21(DE3) (Tiangen Biotech, China), grown in solid LB medium with 50 μg/ml kanamycin. Then, the transformants were induced in ZYP-5052 medium (1% tryptone, 0.5% yeast extract, 0.2% MgSO_4_, 1.25% glycerin, 0.125% glucose, and 10% α-galactose) with shaking (220 rpm) at 20°C for 40 h. After fermentation, the cells were collected by centrifugation (4°C, 8000 × *g*) for 15 min, resuspended in 50 mM phosphate buffer (PB), and then disrupted by sonication. Subsequently, the supernatant was collected by centrifugation (4°C, 9000 × *g*) for 15 min. The crude extract was filtered and purified with Ni^2+^-NTA column in accordance with the instructions (TransGen Biotech, China). Finally, the purified protein was analyzed by SDS-PAGE, and its concentration was determined using a BCA Protein Assay Kit (Thermo Scientific, United States) with bovine serum album (BSA) as the standard. The purified enzyme was then used for further enzyme activity assay and biochemical characterization.

### Enzyme Activity Assay

Enzyme activity assays were performed using the high-performance liquid chromatography (HPLC) method as previously described (Sugano et al., 1994b). The reaction was carried out in a final volume of 100 μl, which contained 50 mM PB (pH 6.0), 308.38 μM NA2, and 0.2 mg/ml of purified enzyme. After incubation at the optimum temperature (30°C) for 10 min, the reaction solution was boiled immediately for 2 min to stop the reaction. Samples were analyzed by HPLC using a Sugar-Pak I column (Waters, United States) and an EDTA calcium disodium solution (50 mg/ml) as mobile phase. The column temperature was 75°C, the flow velocity was 0.5 ml/min, and the detector was a refractive index detector (Agilent 1100, United States). Heat-inactivated enzyme was used as a control. One unit of enzymatic activity (U) was defined as the amount of enzyme that hydrolyzed 1 μmol of NA2 per minute under the assay conditions.

### The pH and Temperature Characteristics Analysis

The optimum pH was determined using 50 mM citric acid-Na_2_HPO_4_ buffer (pH 3.0 to 6.0), 50 mM PB (pH 6.0 to 8.0), 50 mM KH_2_PO_4_-Na_2_PO_4_ buffer (pH 8.0 to 9.0) and 50 mM glycine-NaOH buffer (pH 9.0 to 10.0) at 30°C for 10 min. The optimal temperature was determined in 50 mM PB (pH 6.0) in a temperature range of 20–60°C. For the thermal and pH stability assay, aliquots of enzyme were incubated at 4, 25, 30, and 40°C and pH 6.0 to 9.0 for different times, respectively. Then, the relative enzyme activity was determined according to the method described in the *Enzyme activity assay* section. To examine the effects of series of metal ions (Ba^2+^, Ca^2+^, Co^2+^, Fe^2+^, K^+^, Mg^2+^, Mn^2+^, Na^+^, Zn^2+^, and Ni^2+^) and chemicals (SDS and Na_2_EDTA) on the activity of the enzyme, various metal ions and chemical reagents were added to the 100-μl reaction mixture at a final concentration of 5 mM, and adding buffer without these chemicals or metal ions as control.

### Determination of the Kinetic Parameters of ScJC117

The kinetic parameters of ScJC117 were determined by adding an opportune amount of enzyme (0.3 U purified ScJC117) solution to a substrate solution (30.84–925.14 μM in 50 mM PB, pH 6.0) of NA2. The samples were incubated at 30°C for 10 min and product L-AHG was quantified by HPLC analysis and a standard curve of the product. The *K*_m_ and *k*_cat_ were calculated using the Lineweaver–Burk equation.

### Degradation Pattern Analysis of ScJC117

The degradation products were analyzed by thin-layer chromatography (TLC) and HPLC. Purified ScJC117 (0.6 U) was added to 0.3% A3, A5, A7, A9, and agarose, in 50 mM PB (pH 6.0), and incubated at the optimal temperature for 1 h. Products were analyzed by TLC, loading aliquots of the reaction on silica gel 60 TLC plates (Merck, Germany) and eluting with n-butanol/acetic acid/water (3:2:2, v/v). Then, the reaction products were visualized by soaking the TLC plate in a 10% (v/v) H_2_SO_4_ solution in ethanol, followed by heating at 90°C for 10 min. The reaction carried out with NA2, NA4, or NA6 as substrate was analogously carried out and analyzed by HPLC (as described in the *Enzyme activity assay* section).

### ScJC117 Hydrolysis of NA8, NA10, NA12, and NA14

NA8, NA10, NA12, and NA14 (0.3%) were dissolved in 50 mM PB (pH 6.0) buffer, and purified ScJC117 (0.6 U) was added for hydrolysis at 30°C for 1 h. Products were analyzed by TLC (as described in the *Degradation pattern analysis of ScJC117* section). The AOSs products were analyzed by HPLC with a Superdex 30 increase 10/300 gel filtration column (GE Health, Marlborough, MA, United States) with 5 mM ammonium formate as the mobile phase at a flow rate of 0.4 ml/min; the detector was a refractive index detector (RID) (Agilent, United States). Furthermore, another product, L-AHG, was analyzed by HPLC as described in the *Degradation pattern analysis of ScJC117* section.

### D-Gal and L-AHG Produced by Combining ScJC117 With β-Agarase AgWH50B and AgWH50C

At the beginning of hydrolysis process, dissolve the 1% (w/v) agarose with pH 6.0 50 mM phosphate buffer; then, the agarose should be degraded into NA4 by a β-agarase AgWH50B at 37°C for 12 h, followed by the generation of NA2 catalyzed by an NA2-forming β-agarase AgWH50C at 37°C for 12 h; finally, NA2s are further hydrolyzed to monosaccharides (L-AHG and D-gal) by purified ScJC117 (0.080 U/ml). The crude enzyme activities of AgWH50B and AgWH50C were 0.195 and 0.032 U/mg, respectively. One unit of enzymatic activity (U) was defined as the amount of enzyme that produced 1 μmol of reducing sugar per minute by hydrolyzing agarose under the assay conditions. Results of enzyme amount optimization showed that AgWH50B and AgWH50C were 4.875 and 0.640 U/ml, respectively (Figures 10A–D).

### A7, A9, A11, and A13 Produced by Combining ScJC117 With β-Agarase AgaXa

At the beginning of hydrolysis process, dissolve the 1% (w/v) agarose with pH 6.0 50 mM phosphate buffer; then, the agarose should be degraded into NAOSs by β-agarase AgaXa (3.750 U/ml) at 37°C for 12 h. Then, 0.100 U/ml purified ScJC117 was added to produce AOSs.

## Results

### Sequence Analysis of the and Cloning of ScJC117

**FIGURE 1:**
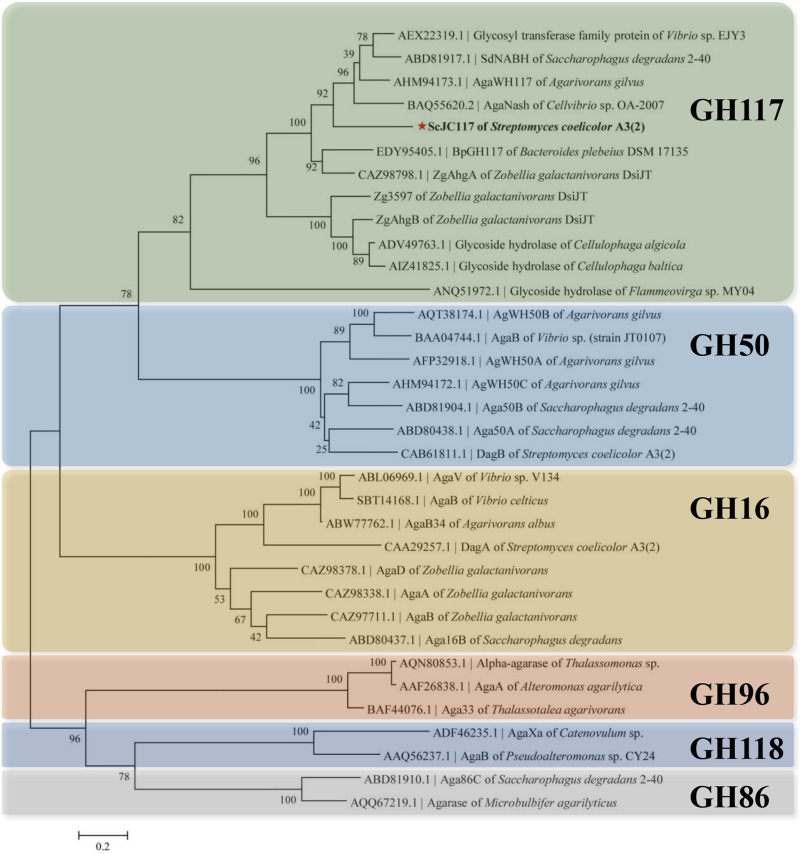
Phylogenetic analysis of agarlytic enzymes from different families. The neighbor-joining tree was obtained using MEGA version 5.0 software. * indicated ScJC117 of this study.

**FIGURE 2:**
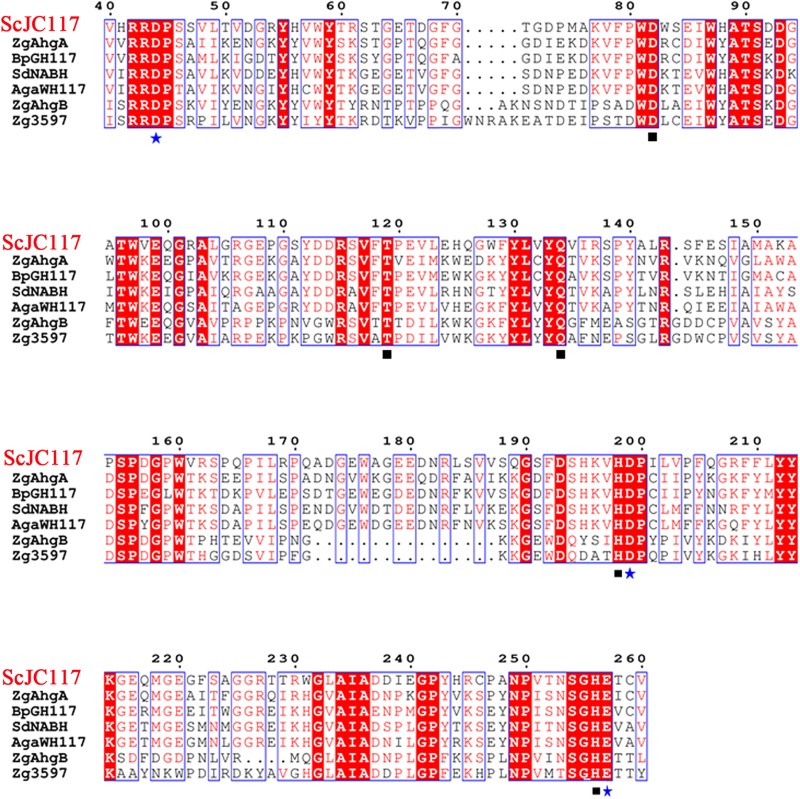
Amino acid alignment of ScJC117. The amino acid alignment of ScJC117; squares (□) denote residues involved in substrate binding, and stars (✩) indicate catalytic residues.

The open reading frame (ORF) of gene *sco3481* from *S. coelicolor* A3(2) encodes an α-NABH protein (GenBank accession No. CAB61805.1), designated as ScJC117, with 370 amino acids. Phylogenetic analysis indicated that ScJC117 belonged to an independent branch of the GH117 family (Figure 1). Protein sequence alignment of ScJC117 showed several highly conserved domains with other known α-NABH (Figure 2), and the acidic amino acids Asp-44, Asp-199, and Glu-257 were identified as those that form the active site (Etienne et al., 2011; Ha et al., 2011). The conserved residues Trp-82, Thr-119, Gln-134, His-198, and His-256 in ScJC117 are presumably involved in the coordination with a NAOS substrate.

**FIGURE 3:**
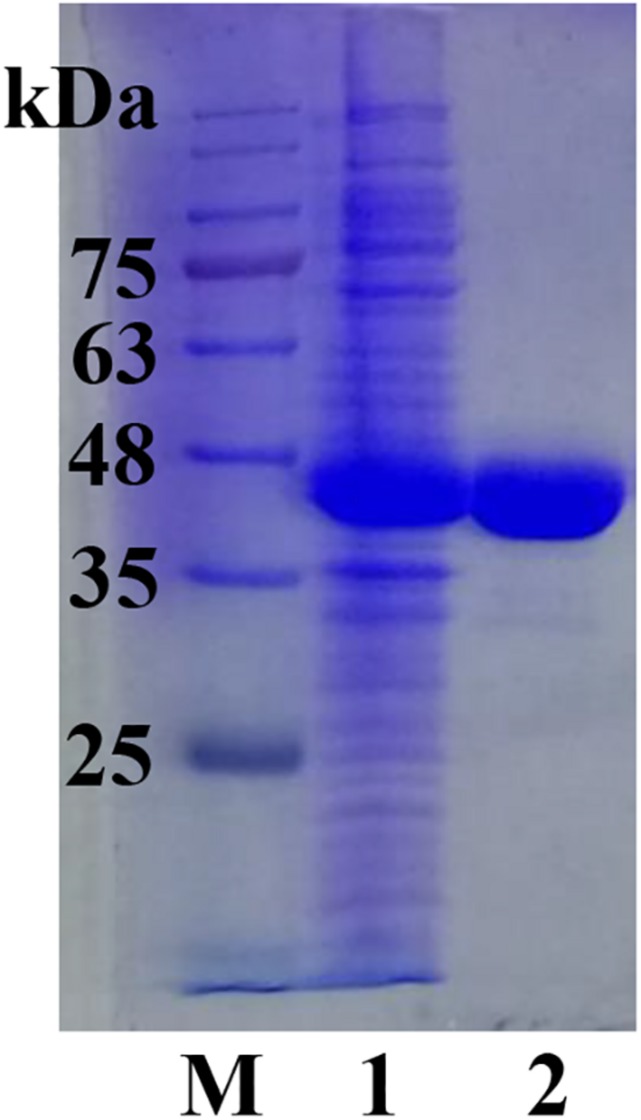
SDS-PAGE analysis of ScJC117. Lane M, protein marker; Lane 1, pET-28a(+) vector-containing control cells; Lane 2, ScJC117 purified by Ni-affinity chromatography.

The full length *sco3481* gene, which encoded the protein ScJC117, was cloned and successfully expressed in *E. coli* BL21(DE3) with pET-28a(+) expression vector. The recombinant protein was purified by Ni-affinity chromatography and detected by SDS-PAGE, which showed one evident band corresponding to 41 kDa, which is similar to its theoretical protein size (Figure 3). The results showed that the recombinant protein could be used for future enzymatic characterization.

### Characterization of ScJC117

Kinetic measurements indicated that *K*_m_ and *k*_cat_ values were 11.57 mM and 0.48 s^–1^, respectively, which are similar to other α-NABHs, such as AgaWH117 and VsNAOSH (Sugano et al., 1994b; Liu et al., 2016).

**FIGURE 4:**
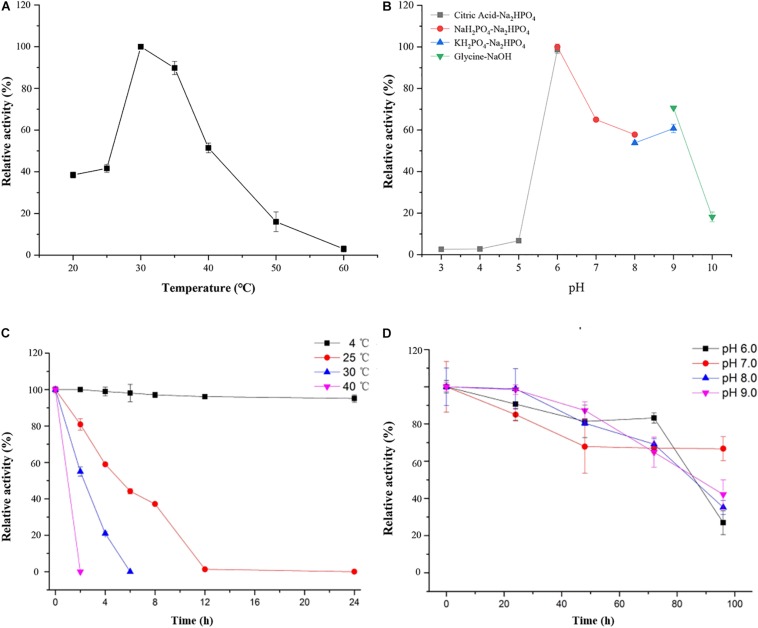
Characterization of ScJC117. Effects of temperature on the activity of ScJC117 **(A)**. Effects of pH on the activity of ScJC117 **(B)**. Effects of pH on the stability of ScJC117 **(C)**. Effects of temperature on the stability of ScJC117 **(D)**. All measurements were performed in triplicate; error bars indicate standard deviation of measurement.

**FIGURE 5:**
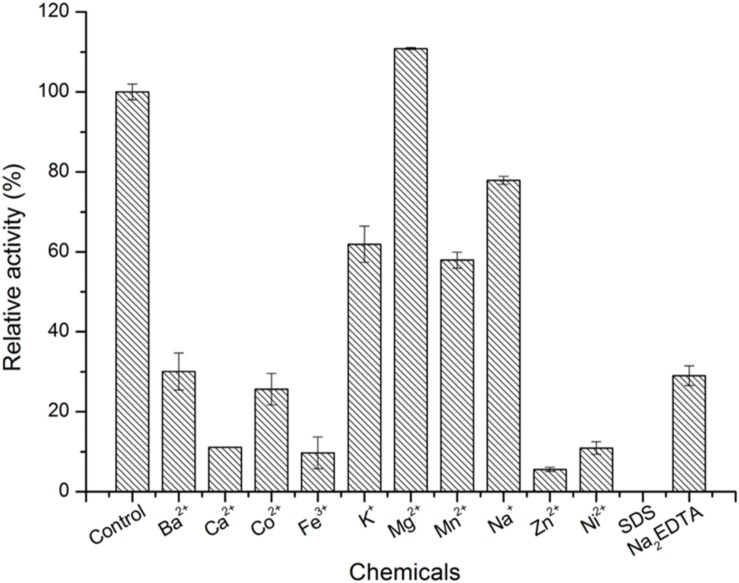
Effects of chemicals on the enzyme activity of ScJC117. The enzyme activity was assayed at 30°C in 50 mM phosphate buffer (pH 6.0). All measurements were performed in triplicate; error bars indicate standard deviation of measurement.

As shown in Figure 4, the optimal temperature and pH of ScJC117 were determined to be 30°C and 6.0, which are the same as BsNAOSH from *Bacillus* sp. and AgaWH117 from *Agarivorans gilvus* WH0801. The enzyme was quite stable at 25°C for 2 h, retaining more than 80% of its activity (Figure 4C), while retaining less than 60% of its activity after incubation for 2 h at 30°C. Meanwhile, ScJC117 showed more than 65% of its initial activity after incubation for 72 h at a pH range from 6.0 to 9.0 (Figure 4D), indicating that ScJC117 could hydrolyze NA2 in a wide pH range. The results in Figure 5 showed that many chemicals including Ba^2+^, Ca^2+^, Co^2+^, Fe^3+^, Zn^2+^, Ni^2+^, and Na_2_EDTA inhibited ScJC117 activity. In addition, the enzyme was completely inhibited by SDS. Conversely, Mg^2+^ slightly increased ScJC117 activity (10%). This is similar to BsNAOSH from *Bacillus* sp. that also had a similar behavior with Mg^2+^ (Suzuki et al., 2002).

### Degradation Pattern Analysis

**FIGURE 6:**
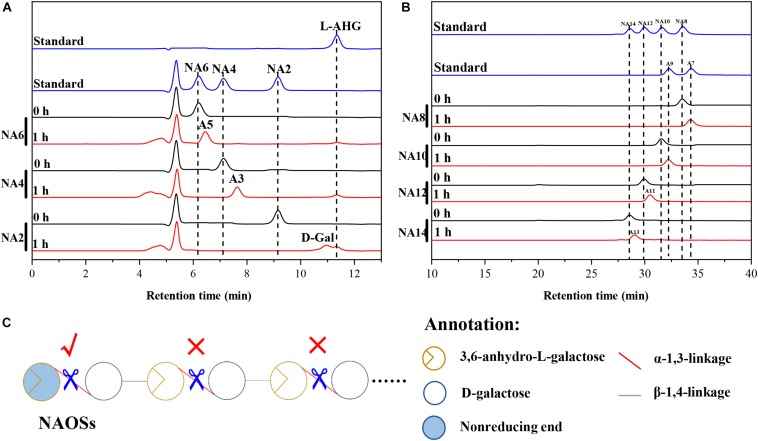
Substrate degradation patterns of ScJC117. **(A)** HPLC analysis of the reaction product of NA2, NA4, and NA6 hydrolyzed by ScJC117. **(B)** HPLC analysis of the AOSs (NA8–NA14) reaction products A7, A9, A11, and A13. NA2, neoagarobiose; NA4, neoagarotetraose; NA6, neoagarohexaose; NA8, neoagarooctaose; NA10, neoagarodecaose; NA12, neoagarododecaose; NA14, neoagarotetradecaose; A7, agaroheptaose; A9, agarononaose; A11, agaroundecaose; A13, agaroundecaose. **(C)** The α-NABHs action mode on NAOSs.

**FIGURE 7:**
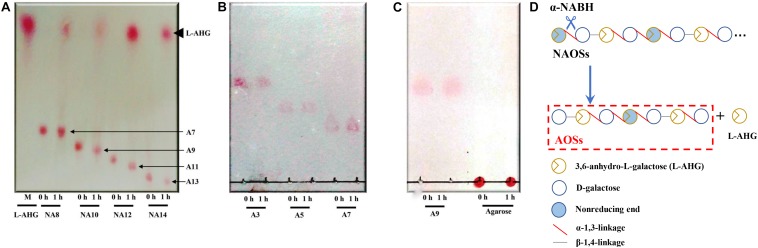
TLC analysis of reaction product of NA8, NA10, NA12, and NA14 **(A)**; A3, A5, and A7 **(B)**; and A9 and agarose **(C)** hydrolyzed by ScJC117. **(D)** Schematic diagram of ScJC117 action mode on NAOSs. NA8, neoagarooctaose; NA10, neoagarodecaose; NA12, neoagarododecaose; NA14, neoagarotetradecaose; A3, agarotriose; A5, agaropentaose; A7, agaroheptaose; A9, agarononaose; A11, agaroundecaose; A13, agaroundecaose.

**FIGURE 8:**
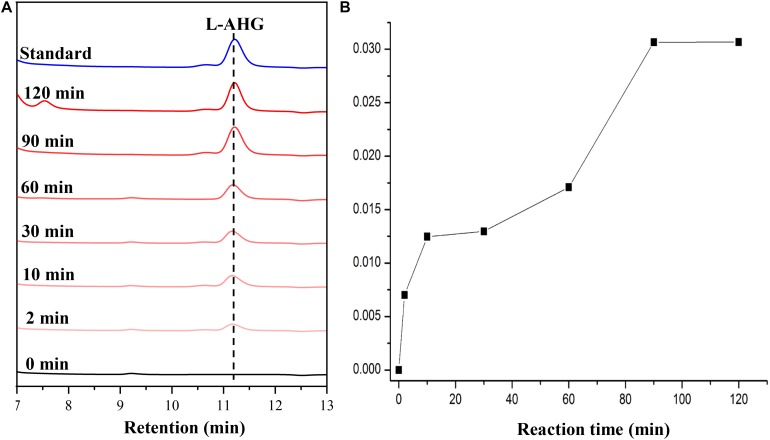
HPLC analysis **(A)** and time course of L-AHG formation **(B)** from NA14 hydrolyzed by ScJC117. NA14, neoagarotetradecaose.

HPLC analysis showed that ScJC117 could act on the NAOSs with different DPs to cut off the non-reducing end L-AHG of NA2, NA4, and NA6 and to produce D-gal, A3, and A5, respectively (Figure 6A). Furthermore, the results of TLC suggested that ScJC117 can also hydrolyze NA8, NA10, NA12, and NA14 into A7, A9, A11, and A13, respectively (Figure 6B), while the L-AHG remained (Figure 7A). Otherwise, Figures 7B,C showed that ScJC117 failed to act on A3, A5, A7, A9, and agarose. Another HPLC analysis was used to further verify the AOSs products from enzymolysis of NA8, NA10, NA12 and NA14; it is indicated that the substrates NA8, NA10, NA12, and NA14 were completely transformed into A7, A9, A11, and A13. To further confirm our result, samples deriving the hydrolysis process of NA14 were analyzed and the L-AHG was quantified by HPLC. As shown in Figure 8A, only one peak with the retention time corresponding to L-AHG was observed after 2 min. The content of L-AHG showed an upward trend with the reaction time (Figure 8B).

## Discussion

α-NABHs not only play an important role in the microbial agar metabolism but also can be applied in food industrial processes for the production of functional monosaccharides and AOSs. In this study, a novel α-NABH, ScJC117, was cloned and characterized. Based on sequence alignment, ScJC117 showed a sequence identity of 57% with three different α-NABHs, AgaWH117 from *A. gilvus* WH0801 (GenBank accession No. AHM94173.1) (Liu et al., 2016), AgaNash from *Cellvibrio* sp. OA-2007 (GenBank accession No. BAQ55620.2), and sdNABH from *Saccharophagus degradans* 2–40 (GenBank accession No. ABD81917.1) (Ha et al., 2011; Ariga et al., 2014), which indicated that ScJC117 has some sequence novelty. Mg^2+^ slightly increased ScJC117 activity and Na_2_EDTA inhibited ScJC117 activity. In a previous study, BpGH117 from the human gut bacterium *Bacteroides plebeius* was discovered having a metal binding site and the crucial ion was Mg^2+^ (Jan-Hendrik et al., 2012). It is deduced that ScJC117 possesses a Mg^2+^ binding site.

**FIGURE 9:**
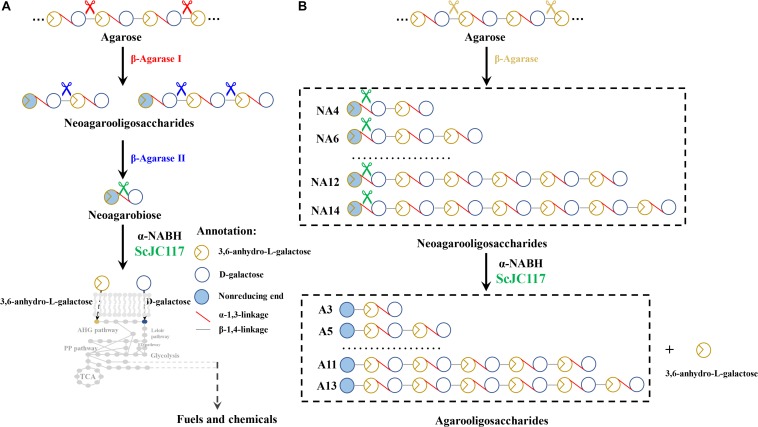
The schematic diagram of agarose degradation by ScJC117 together with agarase **(A)** and producing functional odd-numbered AOSs and L-AHG **(B)**. AOS, agarooligosaccharides; L-AHG, 3,6-anhydro-L-galactose.

**FIGURE 10:**
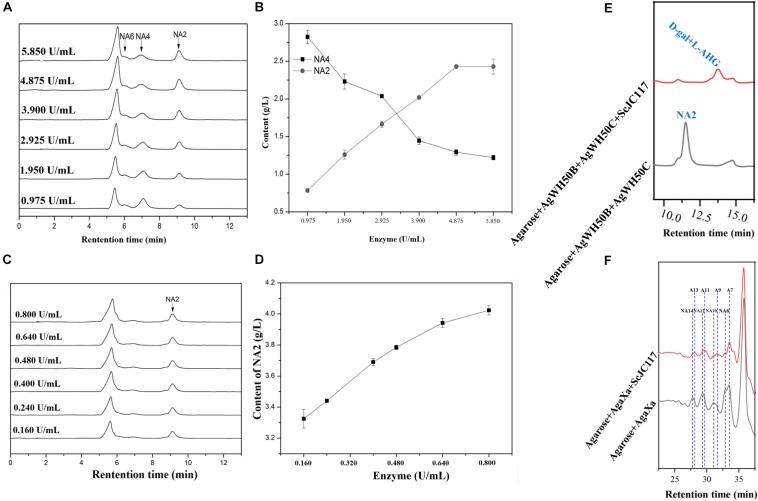
HPLC analysis of enzyme amount optimization of AgWH50B **(A)** and AgWH50C **(C)**, the curves of sugar producing with different amounts of AgWH50B **(B)** and AgWH50C **(D)**. **(E)** HPLC analysis of D-gal and L-AHG producing by combining ScJC117 with β-agarase AgWH50B and AgWH50C. **(F)** HPLC analysis of NA8, NA10, NA12, and NA14 producing by combining ScJC117 with β-agarase AgaXa.

We found that ScJC117 could act on the NAOSs with different DPs to cut off the non-reducing end L-AHG of NA2, NA4, and NA6 and to produce D-gal, A3, and A5, respectively. However, ScJC117 could not act on A3, A5, A7, A9, and agarose. These results revealed that ScJC117 was an exo-type α-NABH; it could act on the first α-1,3-glycosidic bond of NAOS from the non-reducing end (Figure 7D). This result was the same as the previously reported α-NABHs (Ramos et al., 2017), α-NABHs hydrolysis of the first α-1,3-glycosidic bond of NAOS from the non-reducing end but not the second and the others (Figure 6C). Furthermore, ScJC117 shows a good activity toward NAOSs with high DPs (NA8, NA10, NA12, and NA14). In 2017, Ramos et al. summarized the previous reported α-NABH, and the largest NAOS that was used to test the activity is NA8 (Ramos et al., 2017). The PaNABH from *P. atlantica* and CfNABH from *Cytophaga flevensis* were reported to hydrolyze NA2 into D-gal and L-AHG (Day and Yaphe, 1975; van der Meulen and Harder, 1976). Moreover, AgaWH117 from *A. gilvus* WH0801 was able to degrade NA4 (Liu et al., 2016). VsNAOSH from *Vibrio* sp. JT0107 (Sugano et al., 1994a), BsNAOSH from *Bacillus* sp. MK03 (Suzuki et al., 2002), SdNABH from *S. degradans* 2–40 (Lee et al., 2009), and AhgI from *Cellulophaga* sp. W5C could act on NA6 (Ramos et al., 2017). Furthermore, the AhgA from *Z. galactanivorans* could transform NA8 into L-AHG and A7 (Etienne et al., 2011). These results showed that α-NABH could act on NAOSs (NA2, NA4, NA6, and NA8) to produce L-AHG and D-gal or relative AOSs (A3, A5, and A7). It is surprising that not only NA2–NA8 but also NA10, NA12, and NA14 could be hydrolyzed by our enzyme ScJC117. Our result confirmed that α-NABH could indeed hydrolyze NAOSs with the DP higher than 8. To the best of our knowledge, this is the first report that shows that α-NABH can use NAOS with DP higher than 8 as substrate. With NA4–NA14 as substrate, ScJC117 can produce only one monosaccharide, L-AHG, and remained A3–A13. These results suggested that ScJC117 was an important α-NABH to produce bioactive L-AHG and odd AOSs (Figure 9B), especially medium- and long-chain odd-numbered AOSs, such as A7, A9, A11, and A13. Currently, acidolysis was the major method for producing AOSs from agarose because the α-1,3-glycosidic linkages of agarose were preferentially cleaved by acid (Yang et al., 2009; Yun et al., 2016). However, medium- and long-chain AOSs can hardly be obtained from acid hydrolysis of agarose because of the non-specificity and uncontrollability of the acidolysis process (Karlssona and Singh, 1999; Park et al., 2012). Our results suggested that ScJC117 can be explored as a specific tool to produce medium- and long-chain odd-numbered AOSs (A7, A9, A11, and A13) (Figure 10F) combining with a product-specific β-agarase, such as NA8-, NA10-, NA12-, and NA14-forming β-agarase AgaXa from *Catenovulum* sp. X3 (Xie et al., 2013). Compared with acidolysis, the reaction conditions of enzymolysis are much milder, and the ingredients in enzymolysis products are fewer due to the peculiar action mode of β-agarase and α-NABH. For better producing medium- and long-chain odd-numbered AOSs by ScJC117, the technology of enzyme immobilization will be used to improve the temperature stability and achieve reuse in our future work (Liang et al., 2020). Moreover, α-NABH was also crucial in the process of agarose degradation. As shown in Figure 10E, agarose can initially be cut into NAOSs (DPs > 4) by an endo-type β-agarase I (e.g., AgWH50C) (Liang et al., 2017). The NAOSs are further hydrolyzed to NA2 by a GH50-dependent β-agarase II (e.g., AgWH50C) (Liu et al., 2014). Then, α-NABH was used for degrading the NA2 into D-gal and L-AHG (Figure 9A).

## Conclusion

In summary, a novel α-NABH, ScJC117, from *S. coelicolor* A3(2) was isolated and characterized; ScJC117 was an exo-type α-NABH, because it could act on the first α-1,3-glycosidic bond of NAOS from the non-reducing end. With NA4–NA14 as substrate, ScJC117 can produce only one monosaccharide, L-AHG, and remained A3–A13. Therefore, it can be used to prepare L-AHG and functional AOSs, especially medium- and long-chain odd-numbered AOSs, such as A7, A9, A11, and A13, which have potential as functional food additives.

## Data Availability Statement

The datasets generated for this study are available on request to the corresponding author.

## Author Contributions

CJ designed and performed the study and experiments, analyzed the data, and drafted the manuscript. ZL performed the analysis, consulted on experimental design, and revised the manuscript. JS performed and revised the manuscript. XM designed the study and experiments, analyzed the data, and drafted and revised the manuscript.

## Conflict of Interest

The authors declare that the research was conducted in the absence of any commercial or financial relationships that could be construed as a potential conflict of interest.

## Footnotes

1 http://www.ncbi.nlm.nih.gov/

## Abbreviations

A11: agaroundecaose
A13: agaroundecaose
A3: agarotriose
A5: agaropentaose
A7: agaroheptaose
A9: agarononaose
AOS: agarooligosaccharides
D-gal: D-galactose
DNA: deoxyribonucleic acid
DP: degree of polymerization
EDTA: ethylene diamine tetraacetic acid
HPLC: high-performance liquid chromatography
L-AHG: 3,6-anhydro-L-galactose
LB: Luria-Bertani
MEGA: molecular evolutionary genetics analysis
NA10: neoagarodecaose
NA12: neoagarododecaose
NA14: neoagarotetradecaose
NA2: neoagarobiose
NA4: neoagarotetraose
NA6: neoagarohexaose
NA8: neoagarooctaose
NAOS: neoagarooligosaccharides
NTA: nitrilotriacetic acid
ORF: open reading frame
PAGE: polyacrylamide gel electrophoresis
SDS: sodium dodecyl sulfate
TLC: thin-layer chromatography.
